# The life and health challenges of young Malaysian couples: results from a stakeholder consensus and engagement study to support non-communicable disease prevention

**DOI:** 10.1186/1471-2458-14-S2-S6

**Published:** 2014-06-20

**Authors:** SA Norris, H Anuar, P Matzen, JCH Cheah, BB Jensen, M Hanson

**Affiliations:** 1 MRC Developmental Pathways for Health Research Unit, Department of Paediatrics, School of Clinical Medicine, Faculty of Health Sciences, University of the Witwatersrand, Johannesburg PO Box 784314, Sandton, 2146, South Africa; 2 Institute of Health System Research, Ministry of health Malaysia, Suite 55, Setia Avenue, no.2, Jalan Setia Prima s u13/s, Seksyen U13, Setia Alam, 40170 Shah Alam, Selangor, Malaysia; 3 Novo Nordisk Pharma (M) Sdn Bhd, Menara UOA Bangsar, Tower A, Unit A-9-2 & A-9-3, No 5, Jalan Bangsar Utama 1, 59000 Kuala Lumpur, Malaysia; 4 Monash University Sunway Campus, Jalan Lagoon Selatan, 46150 Bandar Sunway, Selangor, Malaysia; 5 Steno Diabetes Center, Niels Steensens Vej 2, DK-2820 Gentofte, Denmark; 6 Academic Unit of Human Development and Health, Southampton General Hospital, Mailpoint 801, South Academic Block, Tremona Road, Southampton, SO16 6YD, UK

## Abstract

**Background:**

Malaysia faces burgeoning obesity and diabetes epidemics with a 250% and 88% increase respectively between 1996 and 2006. Identifying the health challenges of young adults in Malaysia, who constitute 27.5 % of the population, is critical for NCD prevention. The aim of the study was two-fold: (1) to achieve consensus amongst stakeholders on the most important challenge impacting the health of young adults, and (2) to engage with stakeholders to formulate a NCD prevention framework.

**Methods:**

The Delphi Technique was utilised to achieve group consensus around the most important life and health challenges that young adults face in Malaysia. Subsequently, the results of the consensus component were shared with the stakeholders in an engagement workshop to obtain input on a NCD prevention framework.

**Results:**

We found that life stress was a significant concern. It would seem that the apathy towards pursuing or maintaining a healthy lifestyle among young adults may be significantly influenced by the broader distal determinant of life stress. The high cost of living is suggested to be the main push factor for young working adults towards attaining better financial security to improve their livelihood. In turn, this leads to a more stressful lifestyle with less time to focus on healthier lifestyle choices.

**Conclusions:**

The findings highlight a pivotal barrier to healthier lifestyles. By assisting young adults to cope with daily living coupled with realistic opportunities to make healthier dietary choices, be more active, and less sedentary could assist in the development of NCD health promotion strategies.

## Background

Currently, non-communicable disease (NCD) represents 63% of the global burden of disease and is expected to be responsible for 73% of all deaths by 2020. The underlying cause of this NCD epidemic is the increase in lifestyle related risk factors resulting from social and economic transitions [1]. A more concerning trend is that these increases are no longer confined to the elderly population; global trends indicate that NCD-risk behaviours are on the rise among young people and that such lifestyle patterns of dietary and physical activity behaviour persist into older adulthood [2].

From 1996 to 2006, Malaysia saw a dramatic increase in the prevalence of lifestyle-related diseases, including a 250% increase in obesity, 88% increase in diabetes, and 43% increase in hypertension [3]. In the 2011 National Health Morbidity Survey [4], Type 2 diabetes (T2D) prevalence for young adults (18-34 years of age) ranged between 2.1% and 9.4%, and incidence for hypertension, hypercholesterolemia, and especially abdominal obesity was even more alarming with ranges between 8.1-22.2%, 11.3-30.4%, and 19.6-44.7% respectively.

We reviewed the literature and in Malaysia, as with many other low- and middle-income countries (LMICs), rapid urbanisation has resulted in changes in lifestyle. The major determinants of adult obesity and T2D include gender, ethnicity and education. Women were more likely to have greater metabolic disease risk than men. Malays and Indians are at greater risk of obesity and NCDs as compared to Chinese living in Malaysia. Improved education levels are associated with lower risks of metabolic disease [5-13]. Furthermore, as evident elsewhere in the developing world, poor dietary intake (increased convenient food intake), alcohol and tobacco use, and reduced physical activity and increased sedentary behaviour were all associated with greater obesity and NCD risk [10,14-17].

As young people in Malaysia form a significant part of the population, with those in the productive age group of 20-35 years old constituting 27.5% of the population [18], and as they already embody NCD risk factors, it is therefore, critical to identify underlying health challenges facing young adults in Malaysia as part of NCD prevention for improved later adult health. The process of identifying these health challenges requires both a top down (policy makers and other pertinent stakeholders) and bottom up approach (young adults). Thus, the rationale for this study was to convene a heterogeneous group primarily of policy makers and stakeholders, but to also include young adults to ensure their perspectives are captured. Focusing on stakeholders is particularly important as it allows for continued engagement beyond the study to utilise the findings to galvanise support for further developing and implemention of key recommendations. The aim of this study was two-fold; (1) to achieve consensus amongst a diverse group of policy makers, stakeholders, and youth on the challenges impacting the health of young couples in Malaysia, and (2) to engage with stakeholders to formulate a framework linking the factors identified as part of the consensus study with those relevant to NCD intervention development.

## Methods

### Study protocol

Several techniques have been used to achieve consensus on a variety of issues or priorities identified from groups of stakeholders (e.g. Delphi technique, Nominal Group Technique, Focus Groups). The term “consensus” refers to the extent of collective agreement that can be obtained as measured by non-parametric statistics [19]. Intially, the Delphi technique was designed as a systematic process with a panel of experts/stakeholders to achive consensus and assist with forecasting. The principle that underpins Delphi is that consensus through an iterative process will be more accurate [20]. An advantage of the Delphi technique is that it is a relatively efficient method of combining the expertise of a large group of participants to obtain information for planning purposes [21]. The Nominal Group Technique (NGT), which is a structured group engagement to ensure equal participation, assists in facilitating the articulation of a large number of ideas and results, and then to reach agreement on solutions or recommendations that represent the group’s preferences [22]. The advantage of NGT is that it overcomes reticence by some group members to suggest ideas.

The study comprised of two components - consensus and engagement. For the consensus component, the Delphi Technique was utilised with open-ended questions and item ranking to achieve group consensus around life and health challenges that young couples face in Malaysia. Subsequently, the results of the consensus component were shared with the stakeholders in an engagement workshop to obtain input on an NCD prevention framework. For the engagement component NGT was utilised. Ethical approval was obtained from the Medical Research Ethics Council of the Malaysian Ministry of Health (approval code: NMRR-12-1072-14265). Informed consent was obtained from participants in the study.

### Procedures for selecting experts

The identification and recruitment of a group of willing informed participants is a critical step in the Delphi Technique [23]. In selecting and recruiting participants, a Knowledge Resource Nomination Worksheet (KRNW) was prepared to indicate categories of experts. The worksheet was drawn up from the Malaysian National Strategic Plan for Non-communicable disease (NSP-NCD) to identify the most appropriate disciplines and organisations to provide insights into NCD-related issues. The selection of potential participants was centred on three main criteria: (1) relevant expertise; (2) depth of experience; and (3) senior position.

Invitations to participate were sent to the following 19 institutions in Malaysia: Ministry of Health; Ministry of Education; Ministry of Higher Education; Ministry of Youth and Sports; Ministry of Agriculture and Agro-based Industry; Ministry of Transport; Ministry of Information; Ministry of Domestic Trade, Co-operatives and Consumerism; Ministry of Housing and Local Governments; Ministry of Women, Family and Social Affairs; Ministry of Rural and Regional Development, Ministry of Human Resources; Professional and Non-governmental organisations related to health; and Community-based organisations. In addition, a convenient group of lay youth participants involved in a sister qualitative study were also invited. The sampling of a heterogeneous group was purposive as health is inextricably linked to a broad range of determinants, and therefore, inputs from various perspectives can provide richer insights into challenges faced by young adults and couples in Malaysia. Therefore, we felt that including young adults would ensure their perspectives were also captured through both the consensus and engagement process.

### Delphi round 1

It was advised by the Ministry of Health (MoH) that a workshop would be the most suitable and appropriate format given the local context to introduce the study and conduct the first round. An introduction to the Delphi technique and specific instructions were presented and participants had the opportunity to ask clarifying questions. Participants were asked one question and that was to “list in your opinion the most important five life and health challenges that young adults in Malaysia face” and this was completed in private.

### Delphi round 2

After consolidating and summarising Round 1 responses, an e-mail questionnaire was sent to each participant containing the consolidated list of issues and challenges generated from Round 1. Participants were given detailed instructions to: (a) validate the consolidated list of issues by ensuring their round 1 responses were accurately reflected; and (b) select the ten issues that they considered to be most important from the list [24]. Issues that were selected by more than 50% of the experts were identified. This process reduced the list to a manageable size for ranking.

### Delphi round 3 and subsequent rounds

Participants were sent an email asking them to rank the items that had received more than 50% of the votes in Round 2. This process was repeated until consensus was reached. While there are some studies that indicate more difficulty to reach consensus with groups via email than direct interaction [23], the features of convenience and anonymity offers a more robust and independent response as compared to a focus group discussion.

### Stakeholder engagement

Participants were invited to participate in a workshop after the Delphi rounds to discuss the consensus results and engage with researchers to achieve a shared understanding of the most important life and health issues of young adults and how it relates to NCDs.

### Data analysis

The mean rank for each item was calculated and consensus was assessed using non-parametric analysis (Kendall’s Coefficient of Concordance, W). The Kendall coefficient determines whether consensus has been reached, whether the consensus is increasing with subsequent rounds and its relative strength [19]. The coefficient can be interpreted as very weak agreement (0.1), weak agreement (0.3), moderate agreement (0.5), strong agreement (0.7), and unusually strong agreement (0.9).

## Results

### Consensus component

Of the 19 institutions invited, 13 were represented by the 33 stakeholders who consented to participate in the study. From Round 1, 211 responses were noted and filtered for overlap and consolidated by the researchers into a list of 38 distinct items (Table 1 – see Additional file 1).

In Round 2, 26 participants remained in the study (79% of Round 1 participants) and no further attrition was noted. There was no loss to follow-up in the young adult group of participants. Participants in Round 2 were asked to select the ten most important issues from the list of 38 items. Only four items received 50% or more of the votes from the stakeholders.

In Round 3, the four items shortlisted (viz. stressful pace of daily life; high cost of living; work- life balance; financial stress) were then ranked by the participants. High cost of living was agreed by the experts to be the most important challenge faced by couples in Malaysia (mean: 1.96; Table 2 – see Additional file 2). The Kendall Coefficient was found to be 0.22 indicating an overall weak agreement between the experts.

The four items were then sent back to the participants for re-ranking. In Round 4, the mean values for the four items changed, with more participants agreeing on high cost of living being the most important issue (mean: 1.38), leading to a much higher Kendall Coefficient of 0.60 indicating a moderate agreement between experts. As sufficient consensus was obtained, no further rounds were initiated.

### Engagement component

In the stakeholder engagement workshop, the participants deliberated and engaged actively with the researchers to frame their ideas and opinions around the four challenges identified and its relation with NCDs. Two key concepts emerged from the stakeholder engagement. Firstly, a high level of health literacy is needed to better cope with life stress and to change behaviour. Health literacy refers to the cognitive and social skills which determine the motivation and ability of individuals to gain access to, understand and use information in ways which promote and maintain good health [25]. Secondly, the expert panel identified high cost of living as a barrier to healthier lifestyle choices, but also recognised that conversely, having more financial resources also enabled greater access to highly-processed convenience foods and more sedentary lifestyles.

**Figure 1:**
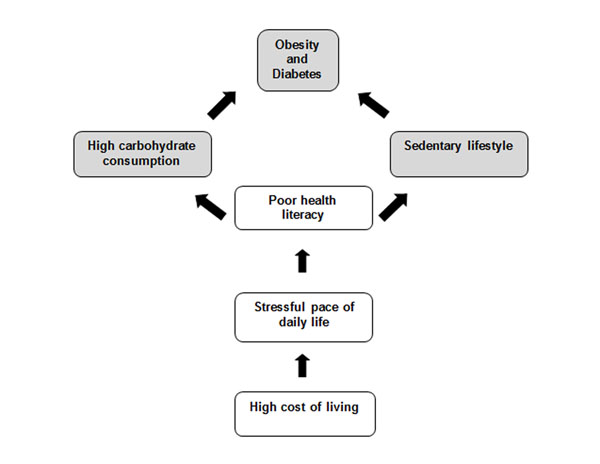
NCD framework linking life stress with poor health literacy and health outcomes

The expert panel concluded that: (1) poor health literacy is the most critical factor leading to conditions such as obesity; (2) high carbohydrate consumption and sedentary lifestyle should be the focal point of health promotion interventions; (3) young adults and married couples in Malaysia prioritise pursuing monetary gains and work over health, and (4) that being relatively young and currently healthy, they are largely ignorant of the risk trajectory associated with unhealthy lifestyle and diet. Participants consolidated ideas into an NCD framework for young married couples in Malaysia as presented in figure 1.

## Discussion

### Young adults and married couples and health

The combination of both the consensus and engagement component was invaluable in deriving a unique NCD framework that can be used for intervention development targeting young adults and couples in Malaysia. In this study we found that life stress was a significant priority and concern. It would seem that the apathy towards pursuing or maintaining a healthy lifestyle among young adults may be significantly influenced by the broader distal determinant of life stress. The high cost of living is suggested to be the main push factor for young working adults towards attaining better financial security to improve their livelihood. In turn, this leads to a more stressful lifestyle with less time to focus on attaining better health literacy and healthier lifestyle choices. The findings in this study resonate with the growing NCD prevalence in LMICs, in that a significant underlying cause of the global NCD epidemic is the increase in lifestyle-related risk factors resulting from social and economic changes. In many countries the increasing impact of globalisation has given momentum to this process [1], and Malaysia is no exception as it continues to urbanise and modernise through its increasing neoliberal economic policy reforms [26].

In addition to the broader structural determinants of economic systems, the apathy in health among the younger generation may also be attributed to the theory of ‘time preference’ in health economics. In particular, given their relatively current healthy physical state, couples may tend have a greater desire to enjoy benefits in the present while deferring any negative effects of doing so [27]. An example of this theory is around smoking, where research highlighted that smokers value future health benefits at a lower level than do non-smokers, preferring current pleasure despite incurring future (discounted) detrimental health effects [28]. The combination of distal determinants and personal time preference creates a vicious cycle that perpetuates poor lifestyle choices and places young couples in Malaysia on a high risk trajectory for developing NCDs.

### Sustaining health capital

The inter-linkage of the four key factors and the NCD risk framework (Figure 1) identified in this study can be broadly associated with the notion of health capital. The theory of health capital encapsulates health demand and investment and its consequence on longevity, and this theory can be applied to both the macro population and micro individual level [29]. Health capital is critical, particularly for young people as sustained or investing in health maintains a healthy biological reserve that may offset ill-health and extend longevity [30]. It is crucial to be aware of the dichotomy between the two sides of the equation, between working lives and financial constraints, versus healthy lives and the financial implications of taking more time to be healthy. Low health capital is associated with the generation and accumulation of health costs throughout the life course, and current health status is a function of the initial level of health and the histories of prior health investments made [29]. Ultimately poor health is likely to result in the passing of less health capital to the next generation as the cost of care escalates. To reduce the burden of NCDs, a greater investment in health capital is needed, which may be achieved by strengthening health literacy in young adults. Health literacy enables young adults to make better health choices and allow young couples to focus more on the quality of life and planning for a family, to give the next generation the best start in life and optimal health capital.

### Study limitations

The composition of the expert panel in this study comprised a heterogeneous group with diverse backgrounds instead of a homogeneous group of experts in a specific field. In a conventional Delphi exercise, a homogenous panel of experts is structured to provide a high level of expertise input [31]. However, we argue that due to the underlying nature of NCDs which relates to a range of determinants across a wide ecology of social, economic, and cultural dynamics, having a heterogeneous group provides the advantage and opportunity of obtaining deeper insights into the complex inter-linkages between these determinants.

## Conclusion

In conclusion, the study derives key information and insights from a broad range of stakeholders which includes policy makers, healthcare professionals, private sector leaders, and the community, and has raised awareness of critical factors that are perceived as influencing lifestyle and health behaviour of young married couples in Malaysia. The combination of the Delphi and Nominal Group techniques was an innovative approach to produce insights from key experts from various backgrounds pertinent to the control and prevention of NCDs in Malaysia. The findings of this study provide crucial information that may assist in the development of NCD policy and health promotion strategies. An important next step is to engage with young adults around the issues of life stress, health capital, and health literacy so as to better understand how Ministry of Health may intervene or support young adults and couples to make healthier dietary choices, exercise more, and be less sedentary. The Jom Mama project is exploring interventions with the Ministry of Health that target young adults/couples prepregnancy to support healthier lifestyle and inturn a healthier future pregnancy.

## Competing interests

The authors declare they have no competing interests.

## Authors’ contributions

SAN, PM, BBJ and MH conceptualised the study. SAN analysed the data. HA and PM collected the data. SAN and JCHC wrote the paper. All authors approved the final version of the paper.

## Supplementary Material

Additional File 1 **Table 1:** List of 38 items from round 1 Click here for file

Additional File 2 **Table 2:** Ranking results from round 3 and round 4 Click here for file

## Declarations

Novo Nordisk funded this study under the partnership with Ministry of Health of Malaysia.

This article has been published as part of *BMC Public Health* Volume 14 Supplement 2, 2014: Responsive health systems: working with the community on control of non-communicable diseases (NCDs). The full contents of the supplement are available online at http://www.biomedcentral.com/bmcpublichealth/supplements/14/S2.
