# Supplementary material for: The life and health challenges of young Malaysian couples: results from a stakeholder consensus and engagement study to support non-communicable disease prevention
Source: BMC Public Health. 2014 Jun 20;14(Suppl 2):S6. doi: 10.1186/1471-2458-14-S2-S6 (PMC4120157; doi:10.1186/1471-2458-14-S2-S6)
Supplement: Additional File 1 — Table 1: List of 38 items from round 1 [file 1471-2458-14-S2-S6-S1.pdf]

Table 1

	**Issues / challenges**	**Explanatory notes**
1	Financial stress	Being concerned about having enough money to have a good life
2	High cost of living	The challenge of dealing with rising prices
3	Finding appropriate housing	It is getting increasingly difficult to find proper housing in Malaysia
4	Work life balance	The difficulty of balancing work/career with private/family life and leisure activities
5	Getting a good job	Concerns related to finding a job that meets one's expectations
6	Fast/stressful pace of everyday life	Everyday life is too busy
7	Poor planning & time management skills	Inability to plan and manage time leads to
8	Marriage life stress	Not being able to cope well with married life due to immaturity and lack of experience
9	Wives earning more than their husbands	Self-explanatory
10	Poor social support	Not having enough social support structures to deal with everyday life challenges
11	Dealing with the influence of in-laws	Being accepted and having a sound, mutually respectful relationship with one's in-laws
12	Dealing with peer pressure (lifestyle, diet)	The impact of peer pressure on life choices around diet, drugs, etc

13	Social disengagement influence of IT	IT poses a barrier to real social relationships
14	Poor parenting skills	Not having the necessary skills and knowledge to be a good parent
15	Pressure to have children	Dealing with social pressure related to starting a family
16	Poor knowledge about the importance of breast feeding	Self-explanatory
17	Infertility / inability to conceive	Self-explanatory
18	Unplanned / early pregnancy	Having children without being sufficiently prepared
19	Poor knowledge about pregnancy	Self-explanatory
20	Having a healthy child	Being concerned about having a healthy child
21	Obesity/overweight	Struggling with weight gain and its negative side effects
22	Hedonistic lifestyle and mind-set	Leading a worry-free lifestyle and not making the right priorities – generally and in relation to health.
23	Sedentary lifestyle	Leading a very sedentary lifestyle; not prioritizing physical exercise
24	Lack of public facilities for exercising	Self-explanatory
25	High consumption of fast foods/snacks	Eating unhealthy foods because this is the cheapest and widely available option
26	Poor dietary habits (general)	Poor eating choices
27	High cost of eating healthier	It is too expensive and inconvenient to eat a

		healthy diet in a consistent way
28	Healthy foods are not tasty	People refrain from eating healthy food because it doesn’t taste good
29	Inconsistent health promotion	Health promotion efforts are not well aligned, leading to confusion around what is right or wrong
30	The effects of advertisements promoting unhealthy foods	Couples’ habits are heavily influenced by marketing tactics and promotions
31	Poor health literacy	Couples lack the basic skills and knowledge to make healthier decisions
32	Challenges around becoming unexpectedly ill	The challenges related to getting an unexpected disease
33	Lack of faith/religion	Neglecting religion leads to lack of direction and guidance in the lives of couples
34	Spousal faith differences	The challenge related to a young spouses not practising their religion in the same way
35	Pollution	The negative effects of an increasingly polluted environment
36	Physical safety	Not feeling safe due to increasing crime rates
37	Sex taboo	Not being able to discuss and resolve matters related to sex
38	Pre-marital sex and STDs	Having to deal with the potential negative effects of having engaged in sex prior to marriage such as STD.
