# Supplementary material for: The life and health challenges of young Malaysian couples: results from a stakeholder consensus and engagement study to support non-communicable disease prevention
Source: BMC Public Health. 2014 Jun 20;14(Suppl 2):S6. doi: 10.1186/1471-2458-14-S2-S6 (PMC4120157; doi:10.1186/1471-2458-14-S2-S6)
Supplement: Additional File 2 — Table 2: Ranking results from round 3 and round 4 [file 1471-2458-14-S2-S6-S2.pdf]

Table 2

	**Mean rank**	
**Items**	**Round 3**	**Round 4**
High cost of living	1.96	1.38
Financial stress	2.04	2.12
Work and social life balance	2.08	2.85
Stressful pace of daily life	3.19	3.65
Kendall Coefficient, W	0.22	0.60
